# Molecular Identification and Antibacterial Activity Analysis of Blue Fox (*Vulpes lagopus*) β-Defensins 108 and 122

**DOI:** 10.3390/ani11071857

**Published:** 2021-06-22

**Authors:** Ling-Ling Li, Tao-Lin Liu, Ping Wu, Nian-Yan Du, Li-Hong Tian, Zhi-Jun Hou

**Affiliations:** College of Wildlife and Protected Area, Northeast Forestry University, 26 Hexing Road, Harbin 150040, China; linglingli0915@163.com (L.-L.L.); taolin.liu@bbctg.com.cn (T.-L.L.); wuping0257@163.com (P.W.); dunianyande@163.com (N.-Y.D.)

**Keywords:** antibacterial activity, molecular identification, vBD108, vBD122, *Vulpes lagopus*

## Abstract

**Simple Summary:**

The blue fox (*Vulpes lagopus*) is an important fur animal in China; its reproductive performance is directly related to the economic benefits of breeding. β-Defensins can protect the male reproductive system from bacterial invasion, maintain the stability of reproductive tract microenvironment and improve semen quality in mammals. Few studies have proposed to investigate the antibacterial effect of *Vulpes lagopus* beta-defensin (vBDs). In this study, we analyzed the antibacterial activity of recombinant vBD108 and vBD122 protein in vitro by an antibacterial activity analysis experiment. Our preliminary results demonstrate that the two vBDs have good antibacterial activity. The blue fox β-defensins may be used as an extender component of the semen diluent to protect semen from bacterial infection.

**Abstract:**

The blue fox (*Vulpes lagopus*), a fur-bearing animal, is an important component of the breeding industry in China. Semen quality is a key factor for the reproductive process and the breeding effectiveness of the farmed blue fox. However, bacterial contamination in semen samples utilized in artificial fertilization is very common. The β-defensins, a class of important antimicrobial peptides in mammals, could protect the reproductive system of male animals from bacterial invasion, maintain the stability of the genital tract microenvironment and improve semen quality. In this study, molecular cloning and bioinformatics analysis were conducted to analyze the protein structure and function of blue fox β-defensin 108 (*Vulpes lagopus* beta-defensin 108, vBD108) and 122 (*Vulpes lagopus* beta-defensin 122, vBD122). To evaluate the bacteriostatic effect of recombinant vBDs (*Vulpes lagopus* beta-defensins) protein, varying concentrations (0, 25, 50, 100, 200 µg/mL) were taken to evaluate the effects on *Escherichia coli* and *Staphylococcus aureus* at different times (0, 2, 4, 6, 8, 12 h). The results showed that vBD108 and vBD122 existed in different forms in protein structure and had antibacterial activity. Both proteins, at 50 µg/mL, had efficacious bacteriostatic activity. This study shows that recombinant vBD108 and vBD122 proteins have good antibacterial activity in vitro. This implies a potential role in improving semen quality and hygienic measures in the process of artificial insemination as an extender of semen dilution with antibacterial activity.

## 1. Introduction

The β-defensins are small cationic peptides rich in cysteine, are from the innate immune system of mammals, and are composed of three disulfide bridges (Cys3-Cys6, Cys1-Cys5, Cys2-Cys4). They typically have a conserved structure of two exons that are translated into the signal peptide and the mature peptide, respectively [1,2]. The β-defensins are mainly localized to the bone marrow, the epithelium and the mucous of the respiratory, genitourinary and gastrointestinal tracts [3,4,5].

The antimicrobial effects of β-defensins can be divided into two types: in the first type, β-defensins act on bacterial cell membranes or walls. Because β-defensins are positively charged and bacterial membranes are negatively charged, when the β-defensins bind to bacterial membranes, the membrane generates pores, and the increased permeability causes the death of the bacteria [6]. Human β-defensin 3 (hBD3) does not directly cause cell membrane perforation, but it inhibits synthesis of the bacterial cell wall by binding to the lipid bilayer, resulting in localized lesions in the cell wall [7]. The other kind of β-defensins act on the interior of bacteria. Some of them can induce bacterial cell agglutination to kill bacteria by phagocytosis of phagocytes, or enter bacteria to inhibit genetic material and protein synthesis [8].

Both native and some synthetic β-defensins have good antibacterial activity. Native hBD3 has considerable activity against *Escherichia coli* and *Staphylococcus aureus* [9], and synthetic canine β-defensin (cBD) can effectively inhibit the activity of *E. coli* and *S. aureus* in vitro [10].

As an important antimicrobial peptide in mammals, β-defensin can not only play a bacteriostatic role on anti-pathogenic microbes but can also participate in the protection of reproductive system tissues from bacterial infection [11,12,13]. Reproductive and urinary tract infections remain an important global public health problem. Male genital tract infections affect reproductive capability and reduce semen quality. Several β-defensins have been identified in the urogenital tract, including human [14,15,16,17], rat [15,18] and dog [10,19]. These β-defensins have been found to have antibacterial activity and play a role in protecting the reproductive tract from microbial infection [20]. In addition, it has been found that porcine β-defensins 1 (PBD1) and 2 (PBD2) at 3 μM, as an antimicrobial in liquid-stored boar semen, have no detrimental effect on sperm viability and motility and are able to control microbial growth effectively [21].

The farmed blue fox (*Vulpes lagopus*), one of the local fur animals in China, is increasing in amount bred every year, and the reproductive performance is directly related to the economic benefits of breeding. The semen used in artificial insemination is the key factor in the reproductive process; however, bacterial contamination of semen used in artificial insemination often occurs in production, causing economic losses to blue fox breeding. Some researchers believe that the addition of β-defensin to commercial extenders for semen can better increase hygienic measures in in vitro fertilization (IVF) and reduce pollution during sperm handling [21]. Previously, we found β-defensins in the testes and epididymides of blue foxes [22]. However, few studies have proposed to investigate the antibacterial effect of β-defensin in blue foxes. The potential antimicrobial function of blue fox β-defensin may play a role in protecting semen from bacterial contamination as an extender component of the semen diluent.

In this study, the molecular cloning and bioinformatics analysis of blue fox β-defensin 108 (*Vulpes lagopus* beta-defensin 108, vBD108) and 122 (*Vulpes lagopus* beta-defensin 122, vBD122) was undertaken to understand the structure and function of vBD proteins, and their antibacterial activity was evaluated by assessing the effect of their action on *E. coli* and *S. aureus*.

## 2. Materials and Methods

### 2.1. Animals and Samples

Five healthy male blue foxes were purchased from Harbin Hualong Blue Fox Breeding Co., Ltd. (Harbin, China). The ages of the blue foxes ranged from 10 to 12 months, and the weights ranged from 11 to 13 kg. Animals were euthanized by intravenous injection with excessive pentobarbital sodium under the International Animal Welfare Law. The testes and epididymides were collected in December 2017 at the College of Wildlife and Protected Area, Northeast Forestry University, Harbin, China. These samples were soaked in RNA preservation solution and stored at −80 °C.

### 2.2. Total RNA Extraction from Tissue Samples

Trizol Reagent kits were used to extract the total RNA from the testis and epididymis samples. Then, the concentration and purity of total RNA was determined by the nucleic acid protein analyzer NanoDrop-2000 (Beijing, China). To confirm the accuracy of the total RNA extraction, the appearance of 18S and 28S ribosomal RNA bands was detected on 1% agarose gel (180 V, 150 mA, 10~15 min).

### 2.3. Reverse Transcription of Total RNA 

The total RNA was reverse-transcribed according to the instructions of the HiScript^®^ⅡQ RT SuperMix for qPCR (+gDNA wiper) kit (Vazyme Biotech, Nanjing, China). The reaction system has two steps as follows: the first step was performed in a total of 20 μL reaction by using 4×gDNA wiper mix 4 μL, with a total RNA 4 μL and RNase free ddH_2_O 12 μL, incubated at 42 °C for 2 min; the second step was performed by adding 4 μL 5×HiScript^®^ II qRT SuperMix II^a^ and 16 μL. The first step of the reaction was conducted at 50 °C for 15 min, then 85 °C for 5 s, and the product of cDNA was stored at −20 °C.

### 2.4. Cloning of vBD108 and vBD122 cDNA

The degenerate primers [23] for the foxes’ β-defensin gene were designated based on the Canine β-defensin sequence (No. XM_003432123 and No. NM_001313788) on the National Center for Biotechnology Information (NCBI) website (http://www.ncbi.nlm.nih.gov/BLAST, accessed on 16 June 2021) using the Primer 5.0 software. The primers were as follows: vBD108, forward primer 5′-GAAGCCKTGTCTGCCTCTG-3′, reverse primer 5′-GATCATTCCTTGGGTGTAG-3′; vBD122, forward primer 5′-GGCAAGTGTTMCAGTAGATT-3′, reverse primer 5′-CTTGGGACAGGGTTATCTT-3′. These were amplified by degenerative polymerase chain reaction (PCR) using the obtained cDNA product template and the degenerate primers. The reaction system was performed in a total of 25 μL as follows: 2 μL of the cDNA template, 1.5 μL of dNTP (2.5 mm/l), 0.3 μL of the Taq enzyme, 0.3 μL of each of the primers (10 pm/μL), 2 μL of 10×PCR Buffer and 18.6 μL of ddH_2_O. The reaction was conducted with a 5 min pre-denaturation at 95 °C, followed by denaturation for 30 s at 95 °C, annealing for 30 s at 53 °C, and extension at 72 °C for 30 s; a total of 30 cycles was used, terminating at 72 °C for 7 min. After agarose gel electrophoresis detection, the resulting PCR products were sent to Jilin Kumei Biotechnology Co., Ltd. (Changchun, China) for sequencing.

### 2.5. Bioinformatics Analysis of vBD108 and vBD122

After sequencing, the cDNA sequences of vBD108 and vBD122 were analyzed by BLAST search at NCBI at https://blast.ncbi.nlm.nih.gov/Blast.cgi, accessed on 16 June 2021, and homology with other species sequences was compared by DNAMAN software. The amino acid sequence and protein physicochemical properties of vBD108 and vBD122 were predicted by online software at http://web.expasy.org/protparam, accessed on 16 June 2021. The SignalP online software at http://www.cbs.dtu.d-k/services/, accessed on 16 June 2021, with default parameters and high stringency search, was used to analyze the signal peptide and phosphorylation sites of amino acids. The three-dimensional structure and space-fill model of vBD108 and vBD122 were predicted using online software at SWISS-MODEL (http://swissmodel.expasy.org/, accessed on 16 June 2021).

### 2.6. Antibacterial Activity

The recombinant proteins vBD108 and vBD122 were chemically synthesized by Shanghai Genscript Biotechnology Co., Ltd., based on amino acid sequences (Table 1).

*Escherichia coli* CGMCC 13344 and *Staphylococcus aureus* CGMCC 18721 (purchased from China General Microbiological Culture Collection Center, CGMCC) were grown to 0.6 OD600_nm_ at 37 °C and 180 r/min overnight in 5 mL Luria-Bertani (LB) liquid medium, respectively, then diluted to 10^6^ CFU/mL with the same medium. Then, 100 µL of recombinant vBD108 and vBD122 protein solution was mixed with 100 μL of diluted bacteria in a polypropylene 96-well microtiter plate, with the control containing only bacteria solution. The final concentrations of recombinant vBD108 and vBD122 proteins were 25, 50, 100 and 200 μg/mL, respectively. These were subsequently cultured at 37 °C for 0, 2, 4, 6, 8 and 12 h. The growth of bacteria was determined by measuring OD600_nm_ (the mean of three independent experiments done in duplicate). The antibacterial activity was indicated by the bacteriostatic rate (Bacteriostatic rate (%) = (OD_control_ − OD_treatment_)/OD_control_ × 100%).

## 3. Results

### 3.1. Bioinformatics Analysis of vBD108 and vBD122

**Figure 1:**
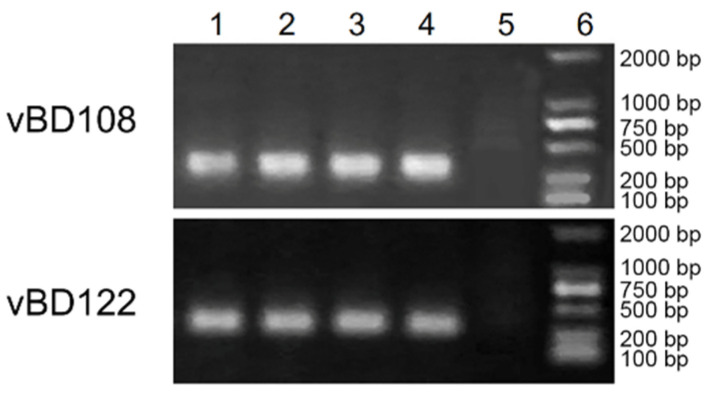
Gene cloning of vBD108 and vBD122 by PCR. (a) 1-4: samples of five blue foxes; 5: control; 6: DNA2000 marker.

**Figure 2:**
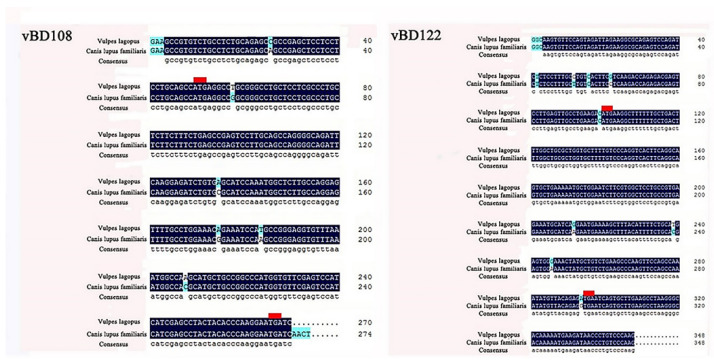
Analysis of vBD108 and vBD122 gene by DNAMAN software. Open reading frames (ORFs) are indicated with start (ATG) and stop (TGA) codons marked in red line in each sequence; blue fluorescence is nucleotide mutation site.

**Figure 3:**
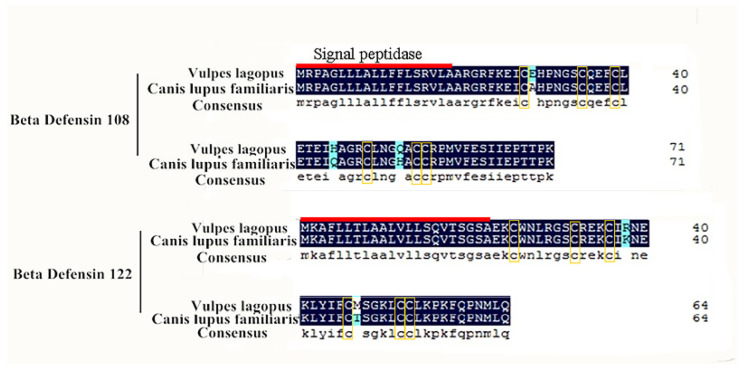
Alignment analysis amino acid sequences of vBD108 and vBD122 in species. Red line marker: signal peptide; blue background: amino acid variation site; yellow box: conserved cysteine residues.

PCR amplification with degenerate primers showed that the cDNA of vBD108 and vBD122 was about 300 bp (Figure 1). In order to be consistent with the naming of canine β-defensin, the cDNA sequences were named vBD108 and vBD122 [19]. The full-length cDNA sequences of vBD108 (270 bp) and vBD122 (348 bp) were obtained using the degenerate primer sequences (Figure 2) and were deposited in the GenBank database (No. MH491012.1 and No. MH491011.1). The entire open reading frames (ORFs) of vBD108 and vBD122 were 219 bp and 198 bp, respectively (Figure 2). Compared with the canine β-defensin gene encoding, DNAMAN analysis revealed that there were five and four nucleotide mutations in the blue fox vBD108 and vBD122 coding regions, respectively (Figure 2). The predicted pre-proproteins of vBD108 and vBD122 were, respectively, composed of 71 and 64 amino acid residues, and the lengths of the signal peptides were 19 and 22 amino acids, respectively. Compared with the amino acid sequence of the cBDs, blue foxes have three and two amino acid mutations, respectively (Figure 3). When aligned, the cysteine spacing patterns of vBD108 and vBD122 with canine β-defensins showed that they share the 6-3-9-5-0 cysteine spacing pattern in their cysteine motifs (Figure 3).

**Figure 4:**
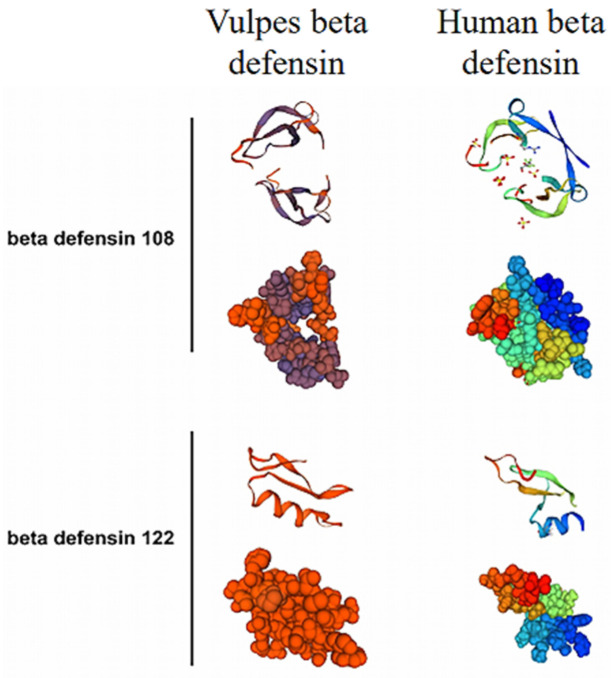
Three-dimensional structure and space-fill model of vBD108 and vBD122 analysis alignment with human beta defensin models.

The amino acid sequence of vBD108 contained 8 positively charged residues (arginine and lysine), and vBD122 contained 11 positively charged residues (arginine and lysine). The three-dimensional structural of vBD108 protein was a homodimer, while vBD122 was a monomer (Figure 4).

### 3.2. Antibacterial Activity

**Figure 5:**
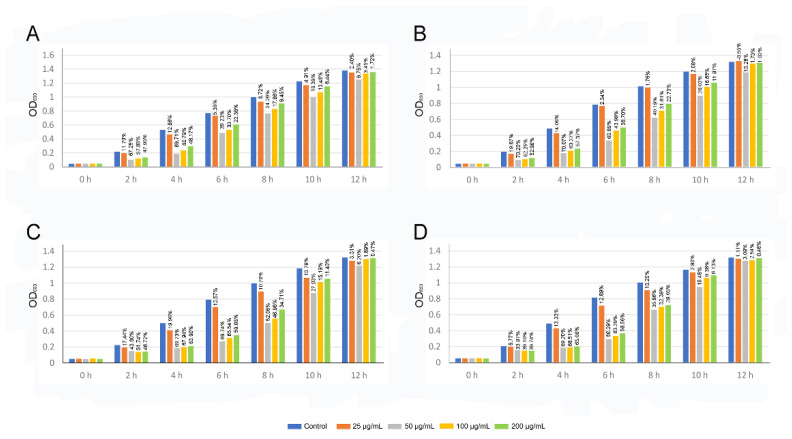
Statistics of OD600nm and bacteriostatic rates in bacterial cultures of vBD108 and vBD122 proteins. (**A**,**B**) Bacteriostatic rates and OD600 nm of *E. coli* and *S. aureus* of vBD108, respectively. (**C**,**D**) Bacteriostatic rates and OD600 nm of *E. coli* and *S. aureus* of vBD122, respectively.

The antibacterial activity of vBDs was measured by the turbidimetric method. The results showed that the vBD108 and vBD122 proteins had a bacteriostatic effect on *E. coli* and *S. aureus*, and they were almost more efficacious at 50 µg/mL (Figure 5). The bacteriostatic effect of vBD108 protein on *E. coli* and *S. aureus* increased at 2 and 4 h; its bacteriostatic rates on *E. coli* were 67.25% and 69.71%, while rates on *S. aureus* were 70.20% and 70.07% (Figure 5A,B). The bacteriostatic rates of vBD122 protein co-cultured with *E. coli* and *S. aureus* are presented in Figure 5C,D; the bacteriostatic effect increases at 4 and 6 h were 69.73%, 69.74% and 69.20%, 68.29%, respectively.

## 4. Discussion

β-Defensins are important components of the innate immune system that promote mammalian immunity and play a significant role in the male reproductive tract [10,12]. Since the initial discovery of the existence of β-defensins, their presence in the epithelial cells of many other organisms, including humans, mice, rats and pigs, has been described, but the effect of β-defensin on blue foxes has not been previously studied. 

The reason why β-defensins kill microorganisms directly is that they have their own special peptide structure. The amino sequence of vBD108 and vBD122 showed that their cysteine distribution patterns were consistent with the cysteine spacing pattern (6-3-9-5-0) of most mammalian β-defensins (Figure 2) (having high antibacterial activity), and they demonstrated the characteristic high concentration of cationic residues (Arg, Lys and His) [10]. The number of positively charged residues in mature peptides of β-defensins is usually 6-14, with an average of 9 [24], which was strongly compatible with the number of cationic amino acid residues of vBD108 (2 Lys, 3 Arg and 3 His; n = 8) and vBD122 (7 Lys, 4 Arg; n = 11). Some researchers have proposed that β-defensin binds and inserts into the cellular membrane of bacteria by the action of high cationic charge density, resulting in the formation of multiple membrane pores, or killing microorganisms by increasing cell penetration through electrostatic interaction [24,25]. This implies that vBD108 and vBD122 may also involve antibacterial activity with positively charged residues.

Furthermore, the prediction results of three-dimensional structure revealed vBD108 could probably exist in the form of a homodimer, while vBD122 might exist in the form of a monomer (Figure 3). There is evidence that the dimeric defensin protein has a stronger binding ability to the membrane proteins of pathogenic microorganisms than monomers during immunization [13,26]. Researchers believe that the increased capacity of HBD3 to form dimers in solution improves the bactericidal activity of hBD3 against *S. aureus* [16]. The homodimers of human alpha-defensin 5 (HD5) could bind to Gram-negative (GN) bacterial inner membranes and result in membrane disruption [27]. These results further infer that vBD108, with a similar three-dimensional structure to HBD3 and HD5, may exert its antibacterial effect by combining with bacterial cell membranes to form pores. In addition, the human β-defensin 2 (hBD2) is monomeric in solution and plays an antibacterial role when it forms oligomers under certain physiological conditions (e.g., high local concentration) [17]. Likewise, vBD122, with a similar three-dimensional structure of hBD2, may play an antibacterial role similar to hBD2. The mechanism of vBD108 and vBD122 taking antibacterial roles needs more research in the future.

Some pathogenic microorganisms, such as *Escherichia coli*, are able to invade and colonize the male reproductive tract, affecting the performance of reproductive organs and the quality of semen, which can cause economic damages in animal breeding [28,29,30]. Some studies have indicated that β-defensins could participate in the nonspecific immune process of the mammalian reproductive tract, such as the first defense line of acute epididymitis caused by bacterial infection [10,15]. This study found that recombinant vBD proteins of vBD108 and vBD122 could inhibit the growth of *S. aureus* and *E. coli*, and the antibacterial activity in vitro was identified in the present study. This was congruent with the results of the amino acid sequence analysis and tertiary structure prediction. This implies that vBD108 and vBD122 may be involved in the defense of innate immune bacteria in the reproductive tract system of male blue foxes, and they could protect the quality of reproductive organs and semen from bacteria.

Microbes affect the semen quality adversely; their presence can decrease sperm longevity within 48 h when collecting and processing during in vitro fertilization (IVF) [31]. Although antibiotics and some recombinant antimicrobial peptides could improve the hygienic measures and control bacterial load during in vitro fertilization, they also have been found to damage sperm motility [21,32]. Meanwhile, overwhelming evidence is accumulating that many recombinant β-defensin proteins, as an agent with antimicrobial activity, can promote sperm motility under in vitro conditions [33,34,35]. The addition of β-defensin 126 (BBD126) in cauda epididymal fluid (CEF) of cattle can significantly improve the motility of bovine sperm [34]. The results of some studies suggested that recombinant human β-defensin 1 (hBD-1) can affect the quality of sperm and improve sperm motility [36,37]. Further, the expression of vBD108 protein in the epididymis of blue foxes with asthenospermia has an apparent difference when compared with healthy samples, suggesting that vBD108 deficiency may related to blue fox asthenospermia [22]. Inhibition of rat epididymal specific β-defensin Bin1b expression resulted in decreased binding of Bin1b to caput sperm and considerably attenuated sperm motility and progressive movement [38]. Therefore, β-defensin, a natural component of the reproduction system, not only has the capacity for antimicrobial activity, but also could potentially improve sperm motility. It was therefore thought that both vBD108 and vBD122 could have a potential positive role as a extenders of semen dilution for artificial insemination of farmed blue fox. 

Although our experimental results found that blue fox β-defensin vBD108 and vBD122 proteins demonstrated antibacterial activity in vitro, we need more experiments to prove that vBD108 and vBD122 proteins can be used as the extender of semen dilution for artificial insemination of the blue fox. We plan to clarify this problem through several aspects in subsequent works. Firstly, vBD antibodies or vBD proteins will be co-cultured with sperm to observe changes in sperm motility. Secondly, the effects of adding the semen extender with vBD protein or with an antibiotic on sperm motility and antibacterial activity will be examined and compared.

## 5. Conclusions

In conclusion, the predicted structures of blue fox β-defensin vBD108 and vBD122 were different, but both demonstrated antibacterial activity against *S. aureus* and *E. coli* in vitro.

## Figures and Tables

**Table 1:** Amino acid sequence for recombinant proteins.

Name	Sequence	Length
vBD108	FKEICEHPNGSCQEFCLETEIHAGRCLNGQACCRPMVFESIIEPTTPKE	49
vBD122	EKCWNLRGSCREKCIRNEKLYIFCMSGKLCCLKPKFQPNMLQR	43

## Data Availability

All datasets generated or analyzed during this study are included in the published article.
